# *Francisella tularensis* Peritonitis in Stomach Cancer Patient

**DOI:** 10.3201/eid1012.040497

**Published:** 2004-12

**Authors:** Xiang Y. Han, Linus X. Ho, Amar Safdar

**Affiliations:** * University of Texas M.D. Anderson Cancer Center, Houston, Texas, USA

**Keywords:** Tularemia, peritonitis, stomach cancer, capecitabine, dispatch *Suggested citation for this article*: Han XY, Ho LX, Safdar A. *Francisella tularensis* peritonitis in stomach cancer patient. Emerg Infect Dis [serial on the Internet]. 2004 Dec [*date cited*]

## Abstract

Tularemia with peritonitis developed in a 50-year-old man soon after diagnosis of stomach cancer with metastasis. The ascites grew *Francisella tularensis* subsp. *holarctica*, which was identified by sequencing analysis of the 16S rDNA. The infection resolved with antimicrobial treatment. Antibodies detected 4 weeks after onset disappeared after chemotherapy-associated lymphopenia.

## Case Study

A 50-year-old man arrived at the emergency department in September 2003 with a 2-day history of high fever (temperature up to 40.8°C), rigors, dry cough, nausea, vomiting, lower abdominal pain, and melena. The patient had recently been diagnosed with signet-ring–cell carcinoma of the stomach with evidence of metastasis to the lung and peritoneum and multiple thoracic and abdominal lymph nodes. Chemotherapy had been planned to start soon.

Physical examination showed fever (temperature 39.7°C), hypotension (96/51 mm Hg), a systolic heart murmur with regular rhythm, and lower abdominal tenderness and rebound. Laboratory examination showed microcytic anemia (hemoglobin 87 g/L), relative neutrophilia (82% of 7.8 x 10^9^/L total leukocytes), and relative and absolute lymphopenia (7% of leukocytes or 0.55 x 10^9^/L). A chest x-ray was normal, as were liver function tests and pancreatic enzymes. A presumptive diagnosis of sepsis with peritonitis was made, and blood and urine were collected for cultures. Empiric cefepime (2 g every 8 h) and tobramycin (one dose 500 mg) therapy was started before hospital admission.

The following day, an esophagogastroduodenoscopy showed cancer ulceration as the source of melena. An echocardiography excluded endocarditis. An abdominal sonogram showed small pockets of ascites in the abdomen and pelvis, and the fluid showed many neutrophils, lymphocytes, and macrophages, consistent with peritonitis. The ascites (5 mL) was also cultured. Despite cefepime treatment, the patient's fever persisted for 36 hours, which prompted a change to imipenem (500 mg every 6 h) and vancomycin (1 g every 12 h). The fever subsided in 1 day, as did the abdominal manifestations. The patient was discharged the following day with further oral gatifloxacin (400 mg four times a day) and amoxicillin/clavulanate (875 mg twice a day) for 10 days.

Anticancer therapy that consisted of radiation to the stomach and daily capecitabine and weekly paclitaxel was begun 5 days after discharge. Two weeks later, at completion of these treatments and the oral antimicrobial drugs, the abdominal lymphadenopathy showed improvement on computed tomography. However, the tumor itself, as well as the lung nodules, remained stable. Additional chemotherapy with three cycles of paclitaxel and carboplatin was started soon afterwards.

Meanwhile, the ascites culture (Bactec Aerobic/F bottle with resins) became positive after 8 days of incubation, and a small gram-negative coccobacillus (strain MDA3270) was isolated. Its fastidious growth and unusual Gram stain features prompted sequencing analysis of the 16S rDNA for identification (*1*). A 586-base pair DNA fragment, amplified by polymerase chain reaction, demonstrated 100% sequence homology with *Francisella tularensis* subsp. *holarctica* (GenBank accession no. L26086, Wilson et al., unpub. data, and AF227312) (*2*). On review, the culture and stain features fit *F. tularensis*. The subspecies was confirmed by the Centers for Disease Control and Prevention (CDC) (Fort Collins, CO). The blood culture remained negative after 7 days of incubation.

The diagnosis of typhoidal tularemia (24 days after onset) led the patient to be further treated with intravenous gentamicin for 2 weeks (120 mg every 8 h), followed by 2 weeks of oral ciprofloxacin (750 mg twice a day). A query of exposure history was also made. The patient was a farmer from northeastern Mississippi and had cut hay in a field infested with rodents 3 weeks before onset. He had traveled from home to Houston for the cancer care. The patient had no history of camping, hunting, or bites by ticks or deerflies. After 6 weeks of anticancer therapy (7 weeks after tularemia), the patient's carcinoembryonic antigen decreased substantially. However, a predominant 6-cm mass in the gastrohepatic ligament region persisted, which raised the question of infection versus cancer. Thus, a percutaneous needle biopsy was performed, and cancerous mucin was demonstrated. Further chemotherapy continued.

A convalescent antibody against *F. tularensis* was detected 4 weeks after onset (titer 1:40, direct agglutination method); however, it disappeared at 3 months after chemotherapy-associated lymphopenia (Figure). Before chemotherapy started, the lymphocyte counts had been 1.0 x 10^9^/L before infection, 0.55–0.40 x 10^9^/L in early infection, and 1.94 x 10^9^/L 8 days postonset. After chemotherapy, however, the counts dropped sharply to 0.07 x 10^9^/L (96% reduction) in 2 weeks. During the remaining weeks of therapy, lymphopenia persisted despite improvement. In contrast, the patient's neutrophil counts were normal to slightly elevated during the entire course.

**Figure:**
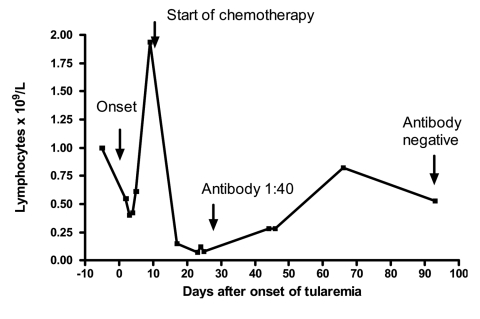
Effect of tularemia and anticancer chemotherapy on the lymphocyte counts and antibody response in a patient with gastric cancer.

## Conclusions

The interest in tularemia and its pathogen, *F. tularensis*, is renewed due to the high bioterrorism potential of the organism, i.e., listed as a category A by CDC (www.bt.cdc.gov). *F. tularensis*, a gram-negative coccobacillus, survives well in the environment and is facultative in infected host cells (macrophages). It has a high infectivity rate and is zoonotic. Human infection occurs mainly in animal handlers and those who are bitten by ticks, deerflies, or both. Airborne and waterborne outbreaks have also been reported (*2**–**4*). From 1990 to 2001, a total of 1,497 tularemia cases was reported to CDC (mean 125 cases per year), with 55% occurring in the states of Arkansas, Missouri, South Dakota, and Oklahoma (*5**,**6*). During those 12 years, however, Mississippi, the home state of our patient, had only one case. In view of the highest disease activity in neighboring Arkansas (324 cases), underreporting of the disease in Mississippi was a possibility in addition to other explanations, such as geographic differences and barriers (Mississippi River).

Tularemia manifests a few clinical forms and, before the antimicrobial era, carried a high fatality rate. The diagnosis of tularemia is often difficult to make, especially for the typhoidal and pneumonic forms. Most cases are diagnosed by serologic tests late in infection or afterwards. In an epidemiologic study of >1,000 cases (*7*), only 11% were diagnosed by isolation of *F. tularensis* from a body source, such as ulcer fluid, blood, lymph node aspirate, and pleural fluid. With improved blood culture methods in the past 2 decades, however, cases of *F. tularensis* bacteremia have been reported (*3**,**8**–**14*). These blood cultures became positive after an incubation period of 3 days to 3 weeks (median 7 days). Most cases were in patients with underlying conditions or diseases, such as old age, alcohol abuse, diabetes mellitus, transplantation, or AIDS. The immunocompromised patients tend to have prolonged infection or die. In a syngeneic bone marrow transplant patient (*15*), the infection presented as a 3-cm solitary pulmonary nodule, and after 6 weeks of antimicrobial treatment, the culture-positive nodule vanished.

Tularemia with associated peritonitis is extraordinary rare. Our patient's peritonitis was likely related to metastatic stomach cancer that had breached the integrity of peritoneum and regional blood vessels and lymph nodes, leading to peritoneal spill of the organism (free or intramacrophage ones). The ascites did contain many macrophages. To combat the infection, neutrophilia developed. Because the patient was severely anemic, absolute lymphopenia developed from normal baseline (Figure). Lymphopenia is generally absent in tularemia, and this patient's response was likely a compromise for neutrophilia. However, the lymphocyte count rebounded a few days later. The patient's response to cefepime therapy was suboptimal in view of the persistence of fever and concurrent isolation of the organism. Streptomycin or gentamicin, not a cephalosporin, is recommended to treat tularemia. Successful treatment with a fluoroquinolone has also been reported in at least 10 recent cases (*11*). The source of infection could not be determined definitively; however, living and working in a farm and the history of exposure to rodent-infested hay were probably important. Recently, landscaping occupation, such as lawn mowing and weed-whacking, is recognized as a risk for exposure (*16*).

The antibody response against tularemia is usually strong, peaking at 2–3 months after onset (*2*). In an outbreak caused by *F. tularensis* subsp. *holarctica*, the peak titer reached 1:256 to 1:8,192 (median 1:1,024) (*2*). In our patient, urgent initiation of the lymphotoxic anticancer chemotherapy blunted the initial response by ablating the antibody-producing lymphocytes. One of the agents, capecitabine, causes lymphopenia in >90% of patients following treatment (*17*). During the 11-week chemotherapy, existing antibodies (titer 1:40) were degraded in the circulation (3–4 half-lives) and became undetectable. Therefore, this case illustrates that, after anticancer chemotherapy, lack of antibody does not exclude an infection that usually elicits antibody response.

*F. tularensis* has four subspecies (biovars): *tularensis*, *holarctica*, *novicida*, and *mediasiatica*, and the first two subspecies are the main causes of tularemia in the United States. *F. tularensis* subsp. *holarctica*, also known as type B or biovar *palaearctica*, is generally less virulent than *F. tularensis* subsp. *tularensis* (type A). Both typhoidal and cutaneous forms have been reported for *F. tularensis* subsp. *holarctica* (*2**,**3**,**9**,**11**,**13**,**14*). For the typhoidal cases, including ours, all nine reported patients recovered, and the median incubation of blood cultures was 9 days (4 days–3 weeks) (*3**,**9**,**11**,**13**,**14*), similar to the 8 days in our ascites culture.

Identifying *F. tularensis* may be difficult because of its rarity and fastidious growth, especially in areas where disease is nonendemic. Our laboratory has been using the 16S rDNA sequencing method to identify mycobacteria and other fastidious organisms. The method is considered to be the single best method to identify bacteria and will likely impact patient care in addition to microbiologic research.
